# Association between cognitive function and dusty weather: a propensity score matching study

**DOI:** 10.1186/s12877-023-04466-0

**Published:** 2023-11-27

**Authors:** Honghui Yao, Zixuan Peng, Xinping Sha

**Affiliations:** 1 Department of Learning, Informatics, Management and Ethics, Karolinska Institute, Stockholm, Sweden; 2 Institute of Health Policy, Management & Evaluation, Dalla Lana School of Public Health, Toronto, Canada; 3 Department of infectious disease. Xiangya Hospital, Central South University, Changsha, 410008 Hunan Province China; 4 Xinagya Changde Hospital, Changde, 415000 Hunan Province China

**Keywords:** Cognition, Air pollutants, Propensity score

## Abstract

**Background:**

With a rapidly aging global population, the health of older adults is a national priority for countries across the world. Dusty weather has been demonstrated to be a potential risk factor of cognitive function among the elderly population. However, there is a paucity of studies exploring the associations between dusty weather and cognitive function among the older in China.

**Methods:**

Data on individual characteristics were obtained from the China Health and Retirement Longitudinal Survey (CHARLS) 2018, whereas data on air pollution were sourced from environmental monitoring stations in China. Cognitive function, including general cognitive function, episodic memory, and linguistic competence, was assessed by self- or informant-questionnaires. We used propensity score matching and linear regression to investigate the relationship between dusty weather and cognitive function. Sensitivity analyses were conducted to test the robustness of the results.

**Results:**

This study included 8,604 participants older than 60 years old. After controlling air pollutant weather, dusty weather was demonstrated to be positively associated with a decline in cognitive function (Informant Questionnaire on Cognitive Decline in the Elderly (IQCODE), 4.0, 95% confidence interval (CI): 3.11, 4.89; Mini‐Mental State Exam (MMSE), 0.63, 95% CI: 0.34, 0.92). Results of sensitivity analysis showed that our research findings are robust.

**Conclusion:**

Older adults living in dusty weather regions suffered a higher level of cognitive impairment, and such adverse effects were more substantial among females compared with their male counterparts. Targeted health interventions to help older adults living in regions where dusty weather occurs frequently are suggested to be proposed.

**Supplementary Information:**

The online version contains supplementary material available at 10.1186/s12877-023-04466-0.

## Background

Cognitive function encompasses the performance of the mental action or processes of perception, learning, memory, understanding, awareness, reasoning, judgment, intuition, and language [1]. It is often measured through parameters like attention span, learning ability, problem-solving, and more [2]. Recent findings indicate that diminished mental processing speed, working memory deficits, and attention inhibition are primary contributors to age-related cognitive decline in older adults [3]. As the world's older population is expanding at an unprecedented rate [4], it becomes crucial to identify factors impacting cognitive function in this demographic.

The impact of age on cognitive function in older adults is multifaceted, with both genetic and environmental factors playing significant roles. Specific inflammatory genes like rs16944 have been shown to

be associated with a generalized cognitive decline, whereas others, such as IL1β SNP rs16944, have been connected solely to memory loss in older adults [5]. Environmental factors, particularly those related to the natural environment, also influence cognitive function through complex mechanisms. For instance, previous studies have shown that road traffic noise [6], distance to natural outdoor environments from a residential address [7], and green space [8] were factors affecting cognitive function in the older. Additionally, This body of research shows that air pollutants such as particulate matter (PM), sulfur dioxide (SO2), and carbon monoxide (CO) can directly or indirectly lead to cognitive impairment [9–12]. However, much of this research concerns chronic exposure, leaving a knowledge gap regarding the effects of transient increases in air pollutants specifically caused by dusty weather.

Dusty weather is a prevalent meteorological condition in regions like Northern Africa, Central Asia, Australia, and China, encompassing blowing dust, dust haze, dust devils, and Haboobs. It is associated with elevated PM, SO2, and CO levels, leading to potential restrictions on outdoor activities [13, 14] and sunlight exposure [15, 16]. Thus, it could be a potential risk factor for cognitive decline, not least because of the inflammatory mechanisms and white matter injury caused by these pollutants [17].

While the effects of long-term air pollutant exposure on cognitive decline have been extensively studied, the influence of transient increases in air pollutants caused by dusty weather remains relatively unexplored [10, 13]. Also, there is a notable lack of studies exploring the impacts of dusty weather on cognitive function in older adults. Most previous research focused on the association between dusty weather and circulatory diseases, respiratory diseases, eye symptoms, cognitive development in teenagers [18, 19], suicide [20], and suicide rates. Moreover, a crucial aspect that remains overlooked in the most existing literature is the need to control air pollution factors potentially caused by dusty weather [18].

In response to these gaps in the existing literature, this study aims to explore the association between dusty weather and cognitive function in older adults, controlling for the effects of air pollutants. The propensity score matching and linear regression were adopted to analyze the impacts of dusty weather on cognitive function the China Health and Retirement Longitudinal Survey 2018 (CHARLS 2018). The study followed the Strengthening the Reporting of Observational Studies in Epidemiology (STROBE) Statement: guidelines for reporting observational studies [21].

## Method

### Data source

Individual characteristic data were extracted from Wave 4 of CHARLS. This wave was chosen because it concentrated on the various dimensions of cognitive function in older populations. CHARLS is a national longitudinal survey launched in 2012. It aims to establish a high-quality public database for individuals aged 45 or older in China [22]. The survey encompasses over 17,000 participants from 450 communities at 150 county-level units (including prefecture-level cities or county-level cities) across 28 provinces of China [22]. The data was collected using a four-stage stratified random cluster sampling method. CHARLS Wave 4 was conducted in 2018, collecting information from 19,816 participants with a response rate of 83.84%. After excluding individuals younger than 60 and samples with missing data, 8604 participants (4032 exposed and 4032 non-exposed) were included in our analysis.

Data for the air quality index (AQI), PM2.5, PM10, SO2, nitrogen dioxide (NO2), ozone (O3), and CO were retrieved from the China National Environmental Monitor Center [23]. The daily average value for each air pollutant in each city was estimated using data from the monitoring station in that city.

### Variables

The study participants were classified into exposure and non-exposure groups based on the frequency of dusty weather events. Frequently, dusty weather occurs in arid and semi-arid regions of central China [24]. As per environmental reports released by the local meteorological administration, participants residing in China, where dusty weather occurred more than three times yearly from 2015 to 2017, were included in the exposure group.

In the older population, cognitive function, including general cognitive function, episodic memory, linguistic competence, delayed recall, and informant reports of cognitive function, was assessed using a range of tests. The general cognitive function was evaluated using the Mini‐Mental State Exam (MMSE) [25] and Telephone Interview for Cognitive Status (TICS) [26], separately. In the MMSE, a score of 25 or higher is deemed normal, with scores below 24 usually indicative of potential cognitive impairment [25]. In the TICS, the normative reference group for interpretation varies based on the purpose of the evaluation and the examinee's age and education level [26]. Episodic memory was evaluated through a recall test involving a list of words presented to the participants, and linguistic competence was measured using the animal fluency test (AFT) - a test quantifying the number of distinct animals a participant could name within a minute [27]. A delayed recall test was performed to measure the delayed recall memory by evaluating the ability to recall specific information after a period of rest or distraction from that information. Informant reports of cognitive function were measured by the Informant Questionnaire on Cognitive Decline in the Elderly (IQCODE) and Community Screening Instrument-Dementia (CSI-D) part 2. A more detailed explanation of these scales and their administration procedures can be found in Supplementary Material 1.

A series of covariates were also considered. These included sociodemographic characteristics, such as age, sex, marital status, minority status, and residential address type; socioeconomic characteristics like education level and location; behavioral factors, such as smoking and drinking; and pre-existing health conditions, including stroke, Alzheimer's disease, Parkinson's disease, and memory problems.

### Statistical analyses

We used multiple imputations by predictive mean matching to impute missing in our primary analysis. Propensity score matching (PSM) was performed using a 1:1 nearest neighbor matching algorithm without replacement with distances determined by logistic regression. Propensity score matching was performed based on the following variables: age, sex, education, marital status, minority, type of residential address, locations, smoking, drinking, and pre-existing health conditions (stroke, Alzheimer’s disease, Parkinson’s disease, and memory problems). The distribution of demographic information before and after propensity score matching is shown in Table 1 and Supplementary Table 1. Comparisons of participant characteristics in the unmatched and matched cohorts, with and without exposure to dusty weather, were performed using the Mann-Whitney U test for continuous variables and the chi-square test for categorical variables. After performing the propensity score, the absolute standardized mean difference in distances between individuals in the exposure and non-exposure group was balanced, with differences less than 0.1 indicative of a good balance. Considering the correlation within cities in the same province, we utilized a linear regression model with clustered standard errors to estimate the association between dusty weather and cognitive function. Additional covariates, such as the city's gross development product in 2018, AQI, PM2.5, PM10, SO2, NO2, O3, and CO, were incorporated into the adjusted regression model. A subgroup analysis was also conducted by gender to account for potential differences in covariates between groups. To verify the robustness of our results, we conducted two sensitivity analyses. The first analysis was based on the pre-matching data, while the second incorporated varying covariates into the propensity score calculation. The estimated effect sizes and 95% Confidence Intervals (CIs) are presented in the results section. All statistical analyses were executed using R programming version 4.2.3.

**Table 1:** Descriptive characteristics of sampled older adults after PSM

	**Exposure group (%)**	**Non-exposure group (%)**	**Absolute standardized difference**
**Demographic information**			
Age, years (median, IQR)	68 (64, 74)	68 (64, 74)	-0.21
Sex*			0.07
Male	2,147 (49.91%)	2,305 (53.58%)	
Female	2,155 (50.09%)	1,997 (46.42%)	
Minority			0.00
Han minority	4,006 (93.12%)	4,001 (93.00%)	
Other	296 (6.88%)	301 (7.00%)	
Marital statues*			0.07
Married or partnered	3,393 (78.87%)	3,269 (75.99%)	
Widowed, separated, or divorced	884 (20.55%)	1,005 (23.36%)	
Never married	25 (0.58%)	28 (0.65%)	
Education			0.04
No Formal Education	1,269 (29.50%)	1,270 (29.52%)	
Less than high school	2,657 (61.76%)	2,621 (60.93%)	
High school	319 (7.42%)	337 (7.83%)	
Post-secondary education	57 (1.32%)	74 (1.72%)	
Type of residential address			0.02
Family housing	4,225 (98.21%)	4,226 (98.23%)	
Nursing home	11 (0.26%)	14 (0.33%)	
Hospital	2 (0.05%)	2 (0.05%)	
Other	64 (1.49%)	60 (1.39%)	
Location			0.04
Central of city/town	823 (19.13%)	856 (19.90%)	
Urban-rural integration zone	248 (5.76%)	273 (6.35%)	
Rural	3,219 (74.83%)	3,158 (73.41%)	
Special zone	12 (0.28%)	15 (0.35%)	
**Pre-existing health conditions**			
Stroke	302 (7.02%)	339 (7.88%)	0.04
Alzheimer’s disease	278 (6.46%)	315 (7.32%)	0.03
Parkinson’s disease	88 (2.05%)	94 (2.19%)	0.03
Memory problems*	342 (7.95%)	414 (9.62%)	0.06
**Behavioral factors**			
Smoking	1,972 (45.84%)	2,058 (47.84%)	-0.02
Drinking	1,360 (31.61%)	1,431 (33.26%)	0.017

^*^p-value < 0.05

## Results

### Descriptive results

After eliminating 9075 samples of individuals under 60 and 1137 unmatched samples, the study was conducted with exposure and non-exposure samples comprising 4302 and 4302 individuals, respectively. The median age in the exposure group was 68 years (interquartile range: 64 - 74 years) compared to 68 years in the non-exposure group (interquartile range: 64 - 74 years). Females constituted 50.1% of the exposure group and 46.4% of the non-exposure group (Table 1). Most participants were of Han minority origin (93.1% exposure; 93.0% non-exposure), resided in family homes (98.2% exposure; 98.2% non-exposure), and lived in rural areas (74.8% exposure; 73.4% non-exposure).

Supplementary Tables 2 and 3 provide a comparative assessment of air pollutants and cognitive function by exposure status. The concentration of all pollutants except O3 in the central dust storm regions was significantly higher than that in other areas. Cognitive function scores—including self-reported general cognitive function, episodic memory, linguistic competence, and informant-reported cognitive function—were restricted growth in the central dust storm regions.

### Association between dusty weather and cognitive function

**Fig. 1:**
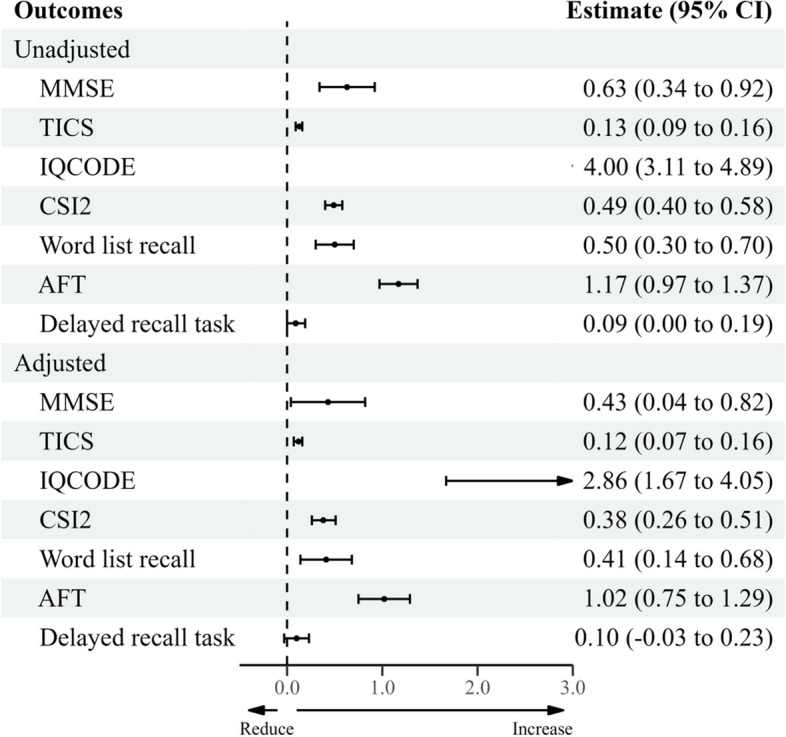
Unadjusted and adjusted associations between dusty weather and cognitive function in the general population

Figure 1 reports the relationship between dusty weather and cognitive function in older adults. Our findings indicated a positive correlation between dusty weather and various cognitive tests: episodic memory (wordlist recall: 0.50; 95% CI: 0.30, 0.70), general cognitive function (MMSE: 0.63; 95%, 0.34, 0.92), and informant-reported cognitive function (CSI2: 0.49; 95% CI: 0.40, 0.58). Despite the positive impact of dusty weather on delayed recall (delayed recall test: 0.09; 95% CI: 0.00, 0.19), this correlation was not statistically significant.

After adjusting for air pollution, we found a statistically significant but weaker correlation between dusty weather and cognitive function, except for the delayed recall test (delayed recall tes: 0.10; 95% CI: -0.03, 0.23).

### Subgroup analysis and sensitivity analysis

**Fig. 2:**
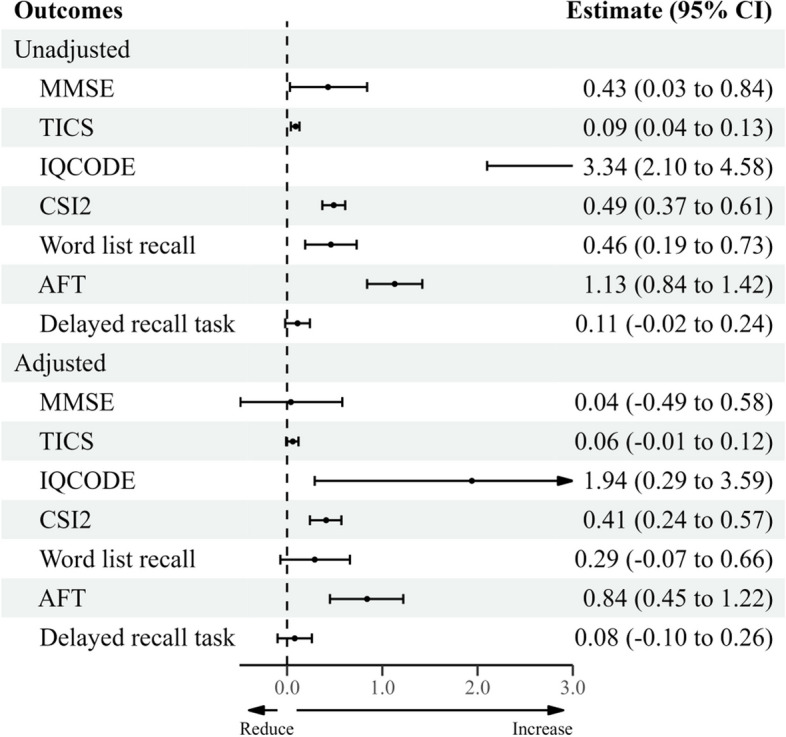
Unadjusted and adjusted associations between dusty weather and cognitive function in males

**Fig. 3:**
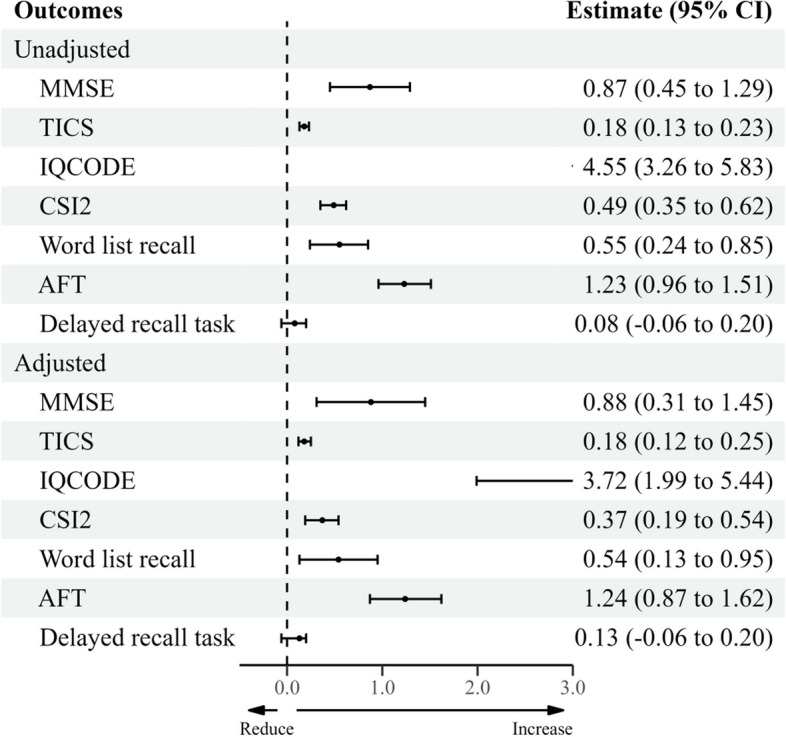
Unadjusted and Adjusted associations between dusty weather and cognitive function in the females

A subgroup analysis was performed with respect to gender, as depicted in Figs. 2 and 3. The data show a significant correlation between dusty weather and cognitive functions in older male adults, but this significance was only observed in models that did not adjust for air pollutants. When these adjustments were made, the associations between most cognitive function indicators (such as the Mini-Mental State Examination and delayed recall) and dusty weather exposure became statistically insignificant.

In contrast, the relationship between dusty weather and cognitive functions in older female adults remained statistically significant in models with and without adjustments for air pollutants, except for the delayed recall test. Interestingly, the impacts of dusty weather on most of the four cognitive function measures were more pronounced in older female adults than in the total sample or male-only group.

The results of the sensitivity analysis can be found in Supplementary Table 4. The findings from both sensitivity analyses align with the primary results, suggesting robustness in our study outcomes.

## Discussion

After controlling for air pollutants, this study revealed that dusty weather is associated with cognitive function in old persons. The positive impacts of dusty weather on cognitive function in older female adults were found to be larger than those in the whole sample or males.

Our study confirmed that dusty weather resulted in cognitive impairment in the older through increased concentrations of air pollutants. Previous research has shown that dusty weather increases the concentration of air pollutants [17], which in turn leads to age-related cognitive impairment [28]. Another potential mechanism is that dusty weather has negative impacts on outdoor activities, which can further lead to cognitive impairment [16]. Besides, dusty weather was also positively associated with an increased risk of cardiovascular diseases that can lead to cognitive impairment [29].

This study also demonstrates the adverse effects of dusty weather on cognitive function were more substantial in females and older adults, which is consistent with a number of previous studies [30, 31]. For instance, a Korean study demonstrated that the relationship between NO2 and cognitive function was stronger in females [30]. A study conducted in Mexico has also shown that the positive effects of PM2.5 on cognitive function in girls were larger than those in boys [31]. This finding may be explained by a stronger female-specific impact of the APOE-ε4, a major genetic risk factor, on age-related cognitive decline [32]. Another possible explanation is that dusty weather or air pollutants can particularly lead to some women-specific diseases like hypertensive pregnancy disorders [33] and menopause [34] that have been reported as risk factors of cognitive decline.

This study suggests further promoting the “Green Great Wall” (GGW), a project established by the Chinese government to build vegetation protective barriers throughout China from the southwestern to the northeastern. A recent study found that during dusty weather in northern China, GGW significantly reduced the concentration of air pollutants, potentially reducing the adverse effects of dusty weather on cognitive function [35].

To the best of our knowledge, this is the first study that explored the potential relationship between dusty weather and cognitive function in older adults in China. This study's evidence could serve as a guide for designing future cognitive intervention programs and an essential reference for other countries facing severe dusty weather issues. Moreover, we controlled the previous diseases that probably influenced the cognitive function in this analysis, increasing our results' validity. Some limitations of this study should be recognized. First, air pollutants were measured at the city level, which may impair the precision of our estimation results. Future studies with individual-level air pollution data can generate more accurate results. Second, limited by data availability, our analysis fails to control other dusty weather-related air pollutants. These air pollutants may exaggerate the relationship between dusty weather and cognitive function. Also, although the correlation between cognitive function and dusty weather was still robust after adjusting for various air pollutants, it is unknown whether our research findings remain consistent after controlling for unobserved additional confounders or mediators. Besides, based on the sampling method, there is probably some oversampling of participants from rural areas and from some provinces in CHARLS. But, because of the lack of individual distribution information in those cities, we can not apply sampling weighting, which may cause potential selection bias. We highly recommend further studies to explore the underlying mechanisms of association. There is abundant room for future research to include more confounding factors to estimate the causal association between dusty weather and cognitive function.

## Conclusions

This study illustrated positive associations between dusty weather and linguistic competence and general cognitive function even after controlling air pollutants. What we found proved the effectiveness of GGW policy from a public health perspective. More interventions should be done to reduce the damage of dusty weather on individuals who live in dusty weather areas.

### Supplementary Information

**Additional file 1:** **Supplementary Table 1.** Descriptive characteristics of sampled older adults before PSM. **Supplementary Table 2.** The comparison of air pollutants in exposure and non-exposure regions. **Supplementary Table 3.** The comparison of cognitive function in exposure and non-exposure groups. **Supplementary Table 4.** Sensitivity analysis – the relationship between dusty weather and cognitive impairment.

**Additional file 2.** 

## Data Availability

The datasets generated and/or analyzed during the current study are available in the China Health and Retirement Longitudinal Survey (http://charls.pku.edu.cn/index/en.html) and China National Environmental Monitoring Center (http://www.cnemc.cn/en/).

## Abbreviations

AFT: Animal Fluency Test
AQI: Air quality index
CO: Carbon monoxide
CHARLS 2018: China Health and Retirement Longitudinal Survey 2018
CSI-D: Community Screening Instrument-Dementia
GGW: Green Great Wall
IQCODE: Informant Questionnaire on Cognitive Decline in the Elderly
MMSE: Mini‐Mental State Exam
NO_2_: Nitrogen dioxide
O_3_: Ozone
PM: Particulate matter
PSM: Propensity score matching
SO_2_: Sulfur dioxide
TICS: Telephone Interview for Cognitive Status
