# Supplementary material for: Association between cognitive function and dusty weather: a propensity score matching study
Source: BMC Geriatr. 2023 Nov 27;23:777. doi: 10.1186/s12877-023-04466-0 (PMC10680218; doi:10.1186/s12877-023-04466-0)
Supplement: Supplementary file 1 — Additional file 1: Supplementary Table 1. Descriptive characteristics of sampled older adults before PSM. Supplementary Table 2. The comparison of air pollutants in exposure and non-exposure regions. Supplementary Table 3. The comparison of cognitive function in exposure and non-exposure groups. Supplementary Table 4. Sensitivity analysis – the relationship between dusty weather and cognitive impairment. [file 12877_2023_4466_MOESM1_ESM.docx]

18,816 individuals with records in CHARLS 2018

Exclude individuals under 60 years old (n = 9075)

9741 individuals (non-exposure: 4598; exposure: 6143)

1:1 propensity score matching

8604 individuals (non-exposure: 4302; exposure: 4302)

**Figure 1 Flow chart of data cleaning**

**Supplementary Table 1 Descriptive characteristics of sampled older adults before PSM**

|  | **Exposure group**  **(%)** | **Non-exposure group**  **(%)** | **Absolute standardized difference** |
| --- | --- | --- | --- |
| **Demographic information** |  |  |  |
| Age, years (median, IQR) | 68 (64, 74) | 67 (64, 73) | 0.73 |
| Sex |  |  | 0.05 |
| Male | 2,317 (50.51%) | 2,955 (48.02%) |  |
| Female | 2,270 (49.49%) | 3,199 (51.98%) |  |
| Minority |  |  | 0.07 |
| Han minority | 4,215 (91.89%) | 5,773 (93.81%) |  |
| Other | 372 (8.11%) | 381 (6.19%) |  |
| Marital statues |  |  | 0.01 |
| Married or partnered | 3,599 (78.46%) | 4,829 (78.47%) |  |
| Widowed, separated, or divorced | 955 (20.82%) | 1,285 (20.88%) |  |
| Never married | 33 (0.72%) | 40 (0.65%) |  |
| Education |  |  | 0.11 |
| No Formal Education | 1,318 (28.73%) | 1,959 (31.83%) |  |
| Less than high school | 2,881 (62.81%) | 3,553 (57.73%) |  |
| High school | 329 (7.17%) | 539 (8.76%) |  |
| Post-secondary education | 59 (1.29%) | 103 (1.67%) |  |
| Type of residential address |  |  | 0.05 |
| Family housing | 4,507 (98.26%) | 6,012 (97.69%) |  |
| Nursing home | 12 (0.26%) | 32 (0.52%) |  |
| Hospital | 3 (0.07%) | 9 (0.15%) |  |
| Other | 65 (1.42%) | 101 (1.64%) |  |
| Location |  |  | 0.12 |
| Central of city/town | 854 (18.62%) | 1,288 (20.93%) |  |
| Urban-rural integration zone | 255 (5.56%) | 478 (7.77%) |  |
| Rural | 3,466 (75.56%) | 4,366 (70.95%) |  |
| Special zone | 12 (0.26%) | 22 (0.36%) |  |
| **Pre-existing health conditions** |  |  |  |
| Stroke | 308 (6.71%) | 567 (9.21%) | 0.15 |
| Alzheimer’s disease | 404 (8.81%) | 386 (6.27%) | 0.10 |
| Parkinson’s disease | 97 (2.11%) | 110 (1.79%) | 0.12 |
| Memory problems | 409 (8.92%) | 501 (8.14%) | 0.03 |
| **Behavioral factors** |  |  |  |
| Smoking | 2,080 (45.35%) | 2,881 (46.82%) | -0.015 |
| Drinking | 1,474 (32.13%) | 1,879 (30.53%) | -0.016 |

**Supplementary Table 2 The comparison of air pollutants in exposure and non-exposure regions**

| **Air pollution indicator** | **Non-exposure regions** | **Exposure regions** |
| --- | --- | --- |
|  | **Mean (SD)** | **Mean (SD)** |
| AQI | 109 (39.92) | 167 (49.13) |
| PM_2.5_ | 81 (32.47) | 127 (40.64) |
| PM_10_ | 129 (47.58) | 183 (64.49) |
| SO_2_ | 23 (10.43) | 51 (33.42) |
| NO_2_ | 50 (15.96) | 63 (18.10) |
| O_3_ | 27 (12.17) | 22 (11.11) |
| CO | 1 (0.29) | 2 (0.66) |

SD, standard deviation; IQR, interquartile range; AQI, air quality index; PM, particulate matter; SO2, sulfur dioxide; NO2, nitrogen Dioxide; O3, ozone; CO, carbon monoxide

All p-values < 0.05

**Supplementary Table 3 The comparison of cognitive function in exposure and non-exposure groups**

| **Cognitive function indicator** | | **Non-exposure group** | **Exposure group** |
| --- | --- | --- | --- |
|  |  | **Mean (SD)** | **Mean (SD)** |
| Self-reported general cognitive function | MMSE | 17 (8.14) | 17 (7.96) |
|  | TICS | 2 (0.89) | 2 (0.91) |
| Self-reported episodic memory | Wordlist recall test | 7 (5.27) | 7 (4.90) |
| Self-reported linguistic competence | Animal naming test | 10 (5.02) | 11 (5.23) |
| Informant-reported cognitive function | IQCODE | 81 (27.40) | 85 (29.50) |
|  | CSI-2 | 7 (2.66) | 8 (2.79) |
|  | Delay recall test | 2 (1.19) | 2 (1.16) |

SD, standard deviation; IQR, interquartile range; MMSE, Mini‐Mental State Exam; TICS, Telephone Interview for Cognitive Status; IQCODE, Informant Questionnaire on Cognitive Decline in the Elderly; CSI-2, Community Screening Instrument-Dementia

All p-values < 0.05

**Supplementary Table 4 Sensitivity analysis – the relationship between dusty weather and cognitive impairment**

|  | **Analysis based on data before matching** | | **Analysis based on different variables included in propensity score** | |
| --- | --- | --- | --- | --- |
| **Cognitive function indicator** | **Unadjusted effect** | **Adjusted effect** | **Unadjusted effect** | **Adjusted effect** |
| MMSE | 0.81 (0.32-1.29) | 0.28 (-0.41-0.98) | 0.78 (0.18-1.38) | 0.42 (-0.47-1.31) |
| TICS | 0.09 (0.03-0.15) | -0.02 (-0.10-0.07) | 0.04 (-0.03-0.12) | -0.07 (-0.17-0.04) |
| IQCODE | 3.87 (2.40-5.34) | 2.22 (0.11-4.33) | 3.54 (1.71-5.36) | 3.24 (0.54-5.93) |
| CSI-2 | 0.42 (0.27-0.57) | 0.28 (0.06-0.49) | 0.39 (0.20-0.58) | 0.27 (0.00-0.55) |
| Wordlist recall test | 0.31 (-0.03-0.64) | 0.22 (-0.26-0.71) | 0.38 (-0.04-0.80) | 0.35 (-0.28-0.97) |
| Animal naming test | 1.20 (0.87-1.53) | 1.07 (0.60-1.54) | 1.05 (0.64-1.47) | 1.04 (0.43-1.66) |
| Delayed recall task | -0.03 (-0.19-0.13) | -0.04 (-0.27-0.19) | 0.01 (-0.19-0.21) | -0.04 (-0.34-0.27) |
